# Chronic Encapsulated Intracerebral Hematoma as an Occasional Finding in Sudden Cardiac Death

**DOI:** 10.3390/healthcare10102053

**Published:** 2022-10-17

**Authors:** Alessandro Feola, Mariavictoria De Simone, Paola Ciamarra, Stefania Sica, Carmela Buonomo, Anna Carfora, Carlo Pietro Campobasso

**Affiliations:** 1 Department of Experimental Medicine, University of Campania “Luigi Vanvitelli”, Via Luciano Armanni 5, 80138 Naples, Italy; 2 Unit of Anatomical Pathology, “Sant’Anna e San Sebastiano” Hospital, Via Ferdinando Palasciano, 81100 Caserta, Italy

**Keywords:** chronic encapsulated intracerebral hematoma, autopsy, intracerebral hemorrhage

## Abstract

Chronic encapsulated intracerebral hematoma (CEIH) is a rare solid mass characterized by the presence of a fibrotic capsule that can present a variety of signs and symptoms due to the mass effect and hydrocephalus. It may be caused by post-traumatic or spontaneous bleeding as related to an adjacent aneurysm, angiomas or neoplasms. Differential diagnosis must be applied in order for it to mimic neoplasm or a vascular malformation. Several cases of CEIH have been reported but only a few of them have an intraventricular localization. A forensic autopsy of a 50-year-old male who died suddenly while driving is discussed. Gross analysis, histology and toxicology were performed and a CEIH of the right lateral ventricle was found in a case of acute coronary death.

## 1. Introduction

Chronic encapsulated intracerebral hematoma (CEIH) is a rare mass characterized by the presence of a fibrotic capsule that histologically resembles the outer capsule of chronic subdural hematoma. It can show a variety of signs and symptoms due to the mass effect and hydrocephalus, depending on the localization. Differently from an intracerebral hemorrhage which shows a sudden onset, CEIH often begins with progressive neurological deficits due to the mass effect [1]. Sometimes, it is associated with vascular lesions such as arteriovenous malformation (AVM), cavernous malformations, capillary telangiectasia, micro-aneurysm, mixed or unclassified angiomas and stereotactic radiosurgery (SRS) for AVM [2,3,4,5]. The size of a CEIH is related to the amount of the hemorrhage, to the obstruction to the blood outflow and the amount of adjacent damaged tissue. However, in most cases the etiology remains unknown [6]. The diagnosis is very challenging, and it is made preoperatively in approximately 20% of cases [1], especially in patients with no clinical history or when other intracranial conditions are present [1,7].

The most common differential diagnosis has to be made with brain tumors (glioblastoma, metastatic brain tumor). Perifocal edema is commonly observed by imaging on both CEIH and tumors. CEIH and brain tumors show also a progressive neurological deficit [1]. Radiologically CEIH can be sometimes misdiagnosed as a cystic brain tumor [8]. In a retrospective review of five patients, Cai et al. (2017) described CT and MRI characteristics of CEIH. On CT imaging, CEIH was reported as almost circular or elliptical cystic lesions surrounded by an enhancement such as brain abscesses [9]. Unfortunately, these findings are not sufficient to confirm a diagnosis of CEIH. On MRI, CEIH has been described as a lesion with avid ring enhancement and intracapsular bleeding [9]. The intracapsular bleeding can show different degrees of T1 and T2 hyperintensity depending on its chronicity. According to other studies [9,10], the ring enhancement with intracapsular bleeding on MRI can be very helpful in the diagnosis of CEIH. Treatment is based on surgical removal of the mass. Two different types of approach are available: (1) craniotomy, and (2) endoscopic approach with aspiration of the hematoma [5,11]. However, it is controversial as to whether the removal of the capsule is necessary, while it has been recommended to prevent a recurrence [11].

A case of CEIH as occasional finding in sudden cardiac death (SCD) is reported. 

## 2. Case Presentation

A 50-year-old male, 105 kg in weight and 180 cm height (BMI: 34,1) with a 30 pack-year smoking history and enalapril therapy for hypertension and no neurological symptoms or pathology, died suddenly while driving. A family history of sudden cardiac death (SCD)—father died at 55 years old for myocardial infarction—was also reported by relatives and the primary care physician. After 72 h, a forensic autopsy was performed.

Histology. For each organ, histological evaluation was carried out according to the guidelines for autopsy investigation of SCD [12]. After fixation in 10% neutral buffered formalin and paraffin embedding, extensive sampling of ventricles and atria was performed. Sections for hematoxylin–eosin staining (H–E) for morphological diagnosis were also examined. 

Toxicology. Femoral blood was analyzed for alcohol (ethanol) and volatiles by head-space gas chromatography coupled with a flame ionization detector (GC/HS-FID). Urine and bile along with brain, liver and kidney were also sampled for the toxicological analyses. All post-mortem specimens were screened for the presence of the main different classes of drugs (pharmaceuticals and illegal drugs) by immunological or chromatographic methods as appropriate. A systematic toxicological analysis (STA) was performed by the LC–MS/MS system (API 3200 triple quadrupole ABI-SCIEX) in multiple reaction monitoring (MRM) mode.

### 2.1. Autopsy Finding—Macroscopic Findings

At autopsy, the heart showed concentric hypertrophy consistent with a history of hypertension. Macroscopically, the heart was 420 g in weight, the transverse size (width) was 13.5 cm. The thickness of the mid-cavity free wall of the right ventricle was 0.5 cm, of the left ventricle was 1.6 cm, and of the interventricular septum (excluding trabeculae) was 1.2 cm. Valves appeared with localized thickening. Coronary sub-occlusion due to atherosclerotic plaque was found at the anterior descending branch of the left coronary. Subtle luminal narrowing and wall thickening was also observed at the right coronary artery soon after the emergence. After cross sectioning of the heart, no clear signs of ischemic myocardial lesions or scarring of myocardial areas were found.

**Figure 1:**
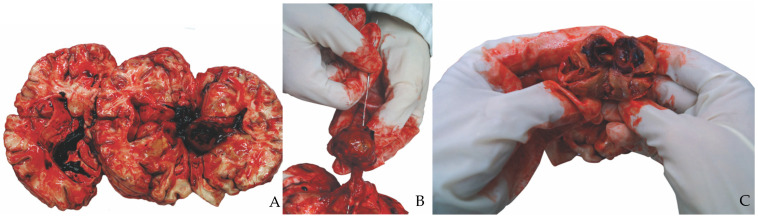
Autopsy findings. (**A**) the brain after sectioning: blood congestion and the tissue mass inside the right ventricle; (**B**,**C**) the tissue mass with fibrous capsule, in the right lateral ventricle, close to right choroidal plexus.

At examination the brain displayed no subdural or subarachnoid hemorrhage. An increased volume of the brain (1390 g in weight) with symmetrical hemispheres was reported. Coronal cuts of the organ were performed according to Ludwig’s dissection of brain. Lateral ventricles were dilated but there was not any transtentorial or subfalcine herniation. Lateral ventricles were found to contain blood clots. As an occasional finding, within the right lateral ventricle, next to right choroidal plexus, there was a round-shaped mass of 2 cm in diameter overlying the floor of the ventricle, near the choroid plexus. The mass was characterized by a hard, thick wall with a fibrous capsule not attached to the surrounding tissue as it was removed easily from the ependymal surface (Figure 1B). The mass showed a band-shaped extension from the main bulk of the mass to the walls of the ventricle. The mass was dissected in situ. The internal part was red brownish in color resembling a hard blood clot (Figure 1A–C). The overall mass was submitted for histological examination. 

### 2.2. Microscopic Findings—Histology

The microscopic appearance of the anterior descending branch of the left coronary atherosclerosis showed a significant narrowing due to proliferating fibrous tissue, calcium, and fat deposition. A diffuse periadventitial lymphocytes–monocytes infiltration was also observed. Stenosis of more than 80% of the lumen was assessed. In this case of acute coronary death the heart showed also hyperosinophilic anucleated myocytes, patches of wavy fibers and contraction band necrosis consistent with early signs of myocardial ischemia.

**Figure 2:**
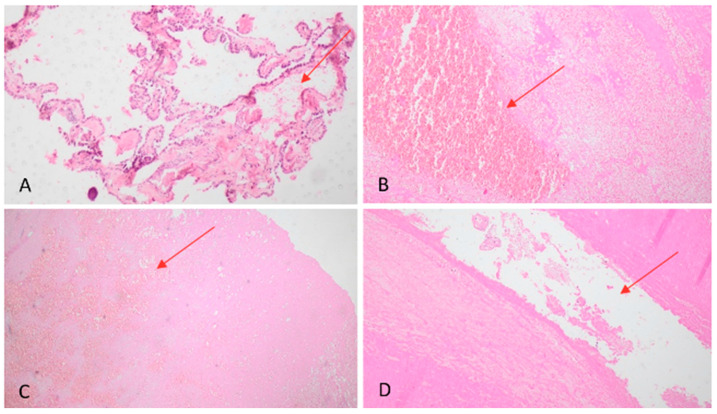
Histology of brain. (**A**) choroid plexus with severe subepithelial edema (red arrow) (H–E ×10); (**B**) intraventricular hematoma in early stage of organization (red arrow) (H–E ×10); (**C**) lysed erythrocytes (red arrow) (H–E ×20); (**D**) flaking choroidal plexus (red arrow) (H–E ×20).

**Figure 3:**
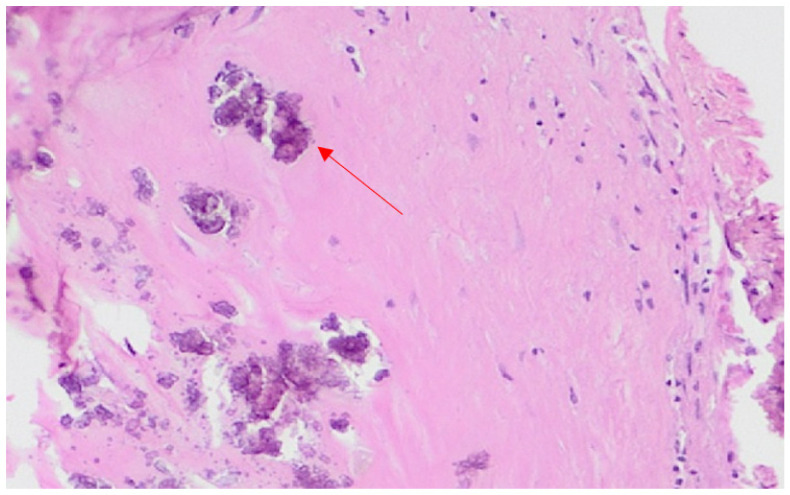
Details of the fibrous capsule characterized by early stage of granulation tissue-type stroma, re-active gliosis, and calcium deposition (red arrow) (H–E ×10).

The microscopic appearance of the brain showed congestion of the choroidal plexus at both lateral ventricles (Figure 2A). Tissue samples taken from the intracranial round-shaped mass showed an organized hematoma with granulation tissue at early stage associated with signs of neovascularization, clusters of fibroblasts, histiocytes and hemosiderin deposits (Figure 2B–D). Reactive gliosis and calcium deposition were found at the hematoma capsule (Figure 3). The capsule was characterized by a thick outer membrane of dense collagen fibers and an inner thin granulation tissue associated with new capillary blood vessels. Within the capsule there was blood clots resulting from old or recent hemorrhages likely due to the rupture of vessels near the inner surface of the fibrous capsule. Based on these histological findings the mass was assessed as CEIH surrounded by a thick, fibrous capsule, consisting of collagenous and granulation tissue.

### 2.3. Toxicological Analysis

Toxicological analysis showed no evidence of drugs or alcohol in bodily fluids and samples of internal organs.

## 3. Discussion

CEIH is a rare entity, and the exact origin is still very challenging for the pathologist [1,3]. It is a disease affecting all ages; the youngest reported patient was 2 months old and the oldest was 80 years old. The mean age is reported to be 41.4 years (standard deviation—sd—20.05 years) and men are supposed to be affected more often than women [13]. Several factors can cause a CEIH. It can be related to an old trauma or a vascular malformation, cavernoma, micro-aneurysm, and venous angioma and it is also described as a complication of SRS for AVM [3,4,14,15,16]. Thrombosis or vascular damage followed by a small intracranial hemorrhage could lead to the formation of a chronic organized hematoma [17] but the mechanism of the capsule formation, the progressive expansions and eventually its resolution is still unclear [6]. CEIH can mimic cerebral tumors, or vascular abnormalities [18]. It can be found in the subcortical region in approximately 80% of cases. However, there are also reports in other areas, including the cerebral deep region, cerebellum, and cerebral ventricle [19]. A few cases of intravascular localization have been reported [19]. The ventricular localization is rare due to the rapidly absorption through the ventricular system and for this reason infrequently undergoes encapsulation. Details of age, sex, presentation, imaging findings, pathology, and outcome of seven cases of CEIH reported in the literature [18,20,21,22,23,24] are summarized in Table S1 in Supplementary Materials. In our case, the hematoma was in the right lateral ventricle with no documented symptomatic mass effect. 

In the literature, several different hypotheses for the CEIH etiology have been proposed among which the formation of a hematoma in the ventricle is correlated to the degree of hemorrhage, the partial obstruction to the outflow of blood and the amount of adjacent damaged tissue. It has been suggested that chronic hematomas are due to initial bleeding of vascular malformations, such as venous angioma o cavernous angioma [14]. Vascular malformations, thrombosed or damaged during hemorrhagic events, could lead also to the formation of chronic organized hematomas [15,17]. Organized hematomas could be the result of a gamma knife radiosurgery (GKR) for cerebral-AVM even though the development of chronic organized hematomas remains unclear [16]. Finitsis et al. (2020) reported an incidence of 1.8% of patients with brain-AVM treated with SRS over a period of 17 years, highlighting that the latency time from radiosurgery to CEIHs diagnosis was 7.7 years (sd 3.7 years) [3]. It has also been suggested that the possible initial cause of CEIH can be minor recurrent bleeding from fragile vessels within radionecrotic brain tissue. As for chronic subdural hematoma, the encapsulated wall of a chronic organized hematoma might reduce the gradual growth with recurrent bleeding from the capillaries in the inner layer of the capsule [25]. Additionally, it has been reported that radiosurgery for AVM can activate the vascular endothelial growth factor (VEGF) pathway, indicating angiogenesis that could lead to chronic organizing hematoma [26]. 

It is worth of mentioning that most bleedings related to intraventricular vascular malformations can reach the cerebrospinal fluid, developing an intraventricular hemorrhage instead of encapsulation [18]. In our case, no blood was found in the cerebrospinal fluid. Histopathologic evaluation of the encapsulated hematoma showed normal architecture of all blood vessels, and there were no dilated thin-walled capillaries or caverns, thereby lacking the prerequisite to classify it in the category of cavernous malformation. Indeed, encapsulated hematoma can be differentiated from cavernous malformation only by histologic examination. In our case, macroscopic examination revealed a well-encapsulated nodular mass measuring 2 cm, red brownish in color which microscopically showed fibrous septa that divided the mass into compartments containing fresh and old hemorrhages without any evidence of tumor growth or AVM. It was the result of a chronic and continuous bleeding from the small vessels’ walls in the intraventricular mass causing a slow growth of the mass with no mass effect. Therefore, it was considered an incidental finding in a case of acute coronary death, although the role of CEIH in a sudden cardiac death cannot be defined with certainty, especially if arrhythmic [27]. 

Clinically, neurological deficits such as symptoms of increased intracranial pressure can appear within weeks or months, with seizures and motor deficits. Before the modern imaging techniques of CT and MRI, skull radiography and ventriculography demonstrated only a vague hyperdensity in the brain parenchyma or ventricular system. CT may show a circumscribed intracranial lesion with mass effect. MRI will demonstrate a well-circumscribed, variably enhancing mass with T2 signal heterogeneity and perilesional edema. Given the high rates of vascular malformations in this case, a vascular imaging technique is recommended, such as MR angiography [20]. Surgery is recommended as treatment to remove CEIH, although spontaneous resolution is sometimes possible [28]. Craniotomy is the most-used approach, followed by the craniectomy endoscopic approach. To avoid further bleeding from the thin capillaries of the capsule, the capsule should be completely drained. If left untreated it can be responsible for the patient’s death. The removal must be performed carefully if a close adhesion of the capsule to vessels of greater caliber and/or vascular malformations is suspected.

## 4. Conclusions

Chronic encapsulated intracerebral hematoma is an uncommon condition that should be reported to contribute to the clarification of etiopathogenesis and the definition of related risk factors. In a case of sudden death, it is important to report findings such as this to understand its role in the mechanism of death.

## Data Availability

The data presented in this study are available on request from the corresponding author. The data are not publicly available due to privacy restriction.

## Supplementary Materials

The following supporting information can be downloaded at: https://www.mdpi.com/article/10.3390/healthcare10102053/s1.
