# Supplementary material for: Chronic Encapsulated Intracerebral Hematoma as an Occasional Finding in Sudden Cardiac Death
Source: Healthcare (Basel). 2022 Oct 17;10(10):2053. doi: 10.3390/healthcare10102053 (PMC9602305; doi:10.3390/healthcare10102053)
Supplement: Supplementary file 1 [file healthcare-10-02053-s001.zip › healthcare-1949608-SM.pdf]

Authors & Years	Age/ Sex	Presentation	Imaging Findings	Pathology	Outcome
Avol & Vogel, 1955 [21]	48, M	Headaches, nausea	Hyperdensity	Well-circumscribed organized hematoma	Complete recovery
Avol & Vogel, 1955 [21]	14, M	Headaches, nausea, vomiting	Hyperdensity	Well-organized and fibrotic blood clot, vascular malformation of choroid plexus	Complete recovery
Lin, et al., 1984 [22]	21, M	Headache, vomiting, palsy of left abducens nerve	Thin ring blush	Old and fresh hemorrages	Complete recovery
Ooba, et al., 2003 [23]	68, F	Gait disturbance, hemiparesis	T1 isointense, T2 mixed intensity, heterogeneous contrast enhancement	Organized hematoma, granulomatous tissue	Complete recovery
Kechagias, et al., 2010 [24]	30, M	Gait disturbance, fever, palsy of N. III, VI, VII, photophobia	High signal intensity on T1 and T2 weighted images	Granulation tissue, neovascularization	Complete recovery
Dey, et al., 2011 [18]	47, F	Apraxia, neglect	Multilobulated lesions, calcifications and hemorrage	Hematoma of different ages	Complete recovery
Wetzel, et al., 2018 [20]	14, M	Headache, blurred vision, nausea, vomiting	Isodense mass with surrounding hyperdensity	Organized hematoma with fibroblasts	Complete recovery

Table S1. Summary of cases of CEIH with an intraventricular localization.
